# Eye tracking cognitive load using pupil diameter and microsaccades with fixed gaze

**DOI:** 10.1371/journal.pone.0203629

**Published:** 2018-09-14

**Authors:** Krzysztof Krejtz, Andrew T. Duchowski, Anna Niedzielska, Cezary Biele, Izabela Krejtz

**Affiliations:** 1 Department of Psychology, SWPS University of Social Sciences & Humanities, Warsaw, Poland; 2 School of Computing, Clemson University, Clemson, SC, United States of America; 3 Interactive Technologies Laboratory, National Information Processing Institute, Warsaw, Poland; State University of New York Downstate Medical Center, UNITED STATES

## Abstract

Pupil diameter and microsaccades are captured by an eye tracker and compared for their suitability as indicators of cognitive load (as beset by task difficulty). Specifically, two metrics are tested in response to task difficulty: (1) the change in pupil diameter with respect to inter- or intra-trial baseline, and (2) the rate and magnitude of microsaccades. Participants performed easy and difficult mental arithmetic tasks while fixating a central target. Inter-trial change in pupil diameter and microsaccade magnitude appear to adequately discriminate task difficulty, and hence cognitive load, if the implied causality can be assumed. This paper’s contribution corroborates previous work concerning microsaccade magnitude and extends this work by directly comparing microsaccade metrics to pupillometric measures. To our knowledge this is the first study to compare the reliability and sensitivity of task-evoked pupillary and microsaccadic measures of cognitive load.

## 1 Introduction

Cognitive Load Theory (CLT) [1] plays an important role in Human-Computer Interaction (HCI) research. There is a pressing need for a non-invasive measure of individuals’ cognitive load, as it can guide designers of interactive systems to avoid overloading users. Measurement of cognitive load could allow a system to respond appropriately, potentially either by toning down or ramping up the level of task difficulty e.g., as in e-learning systems [2], or by adapting mission-critical systems to the user’s cognitive state [3]. Examples of its use include a wide range of applications, including surgery [4], flight safety [5], human-centered design, human cognition modeling, usability, and multimedia learning [6, 7]. A reliable, and possibly real-time, measurement of cognitive load is thus highly desirable. However, due to a lack of its reliable measurement, only a weak link exists between Human-Computer Interaction and Cognitive Load Theory [8].

Yuksel et al. [8] list the predominant measurement methods in CLT studies as self-reporting, the dual-task paradigm, and physiological measures (see also [9]). Eye tracking, a type of physiological measurement, may offer the greatest potential for a reliable, non-invasive estimate of cognitive load [10]. One eye-tracked measure recorded as a matter of course is pupil diameter, which has been exploited as a measure of cognitive load, termed the Task-Evoked Pupillary Response (TEPR) [11].

The pupil diameter’s indication of cognitive load can be traced back to Hess and Polt [12], who demonstrated the relation between pupil dilation and task difficulty (pupil diameter increases with problem difficulty). Kahneman and Beatty [13] suggested that pupil diameter provides a “very effective index of the momentary load on a subject as they perform a mental task.” Generally, the idea that pupil size can be considered as a valid index of “cognitive load” has been widely reported in many different contexts related to cognition; for more extensive reviews, see Beatty [14] and van der Wel and van Steenbergen [15].

Duchowski et al. [16] note the pupil’s sensitivity to a number of factors unrelated to cognitive load, including ambient light [17] and off-axis distortion [18]. As an alternative to the TEPR, they offer the Index of Pupillary Activity (IPA) and show its sensitivity to task difficulty in a replicated study originally designed by [19].

In this paper, we offer an alternative estimate of cognitive load based on measurement of *microsaccades* during mental calculation tasks. Unlike either of the earlier two studies [16, 19], we compare microsaccadic metrics to measures of pupil diameter, namely the averaged difference in pupil diameter with respect to inter- or intra-trial (averaged) baseline. We suggest that measurement of microsaccadic activity is a viable alternative to pupil-based measures and to the IPA. Because microsaccades can be detected within the streaming eye tracker raw point data, **p**_*t*_ = (*x*(*t*), *y*(*t*)), and are not susceptible to influence from ambient light, they offer good potential for non-invasive, real-time measure of cognitive load.

## 2 Related work: Eye tracking

Because eye trackers report pupil diameter as a matter of course, there is renewed interest in using them in lieu of a pupillometer for the purpose of estimating cognitive load. There are two somewhat divergent assumptions regarding the relationship between pupil diameter and cognitive load:

- pupil diameter should be measured with respect to the average pupil diameter measured during a baseline trial (termed here *inter-trial change in pupil diameter—BCPD*);
- pupil diameter should be measured with respect to the average pupil diameter measured during a baseline measurement made at the beginning of each trial (termed here *intra-trial change in pupil diameter—CPD*).

Both baseline-related measures are difference scores, where the baseline pupil size is subtracted from the post-baseline pupil size. Measurement of the change in pupil diameter in relation to its baseline is performed due to the assumed correspondence between its *tonic* and *phasic* components. The TEPR is assumed to correspond to the pupil’s phasic response, while tonic pupil diameter corresponds to its baseline diameter [5]. The pupil diameter baseline measurement is thus taken with the hopes of recording the tonic pupil diameter, its sustained component of the pupillary response. The pupil’s phasic response refers to a transient component of the pupillary response, expressed as dilation relative to the baseline. A few examples of how eye-tracked pupil diameter has been used for estimation of cognitive load are given below.

An early experiment using an eye tracker (a 50 Hz Applied Science Labs model 1994) to measure pupil diameter was conducted by Hyönä et al. [20]. In their experiment on language tasks of different complexity, they used measurements of pupil diameter compared across tasks. They referred to this as global processing (of pupil diameter). Klingner et al. [21] in their review of eye trackers used for cognitive load estimation refer to this type of pupil diameter measurement as coarse, time-aggregated data processing, i.e., an aggregated measurement of pupil diameter over a long period of time. In contrast to coarse measurement, Klingner et al. [21] also suggest pupil measurement following a 2 sec delay after stimulus onset (e.g., intra-trial change in pupil diameter). They advocate detailed timing and evaluation of short-term pupillary response.

Kruger et al. [22] consider task difficulty when viewing video with or without subtitles. They use percentage change in pupil diameter as an indicator of cognitive load. They advocate the use of baseline pupil diameter, e.g., measured when reading instructions, or during some other introductory tasks prior to the test trial(s), i.e., inter-trial measurement.

Chen & Epps [23] used a 12 second task window during which they computed the average pupil diameter, and then subtracted the average pupil diameter recorded during a 0.5 second baseline time window. They thus used a form of intra-trial baseline subtraction, taking care to examine variations in stimulus background variations. Their results conformed with previous studies of [14], [24], and [25].

Kiefer et al. [26] examined pupil diameter during visual exploration of common web maps under six different task demands, including free exploration, visual search, polygon comparison, line following, focused search, and route planning. By considering changes in mean pupil diameter across tasks, their pupillometric inter-trial measure is similar to that of Hyönä et al. [20], who treated a separate trial as baseline for pupil diameter comparison.

We test both forms of intra- and inter-trial pupil diameter difference measurements and refer to them as Change in Pupil Diameter (CPD) and Baseline Change in Pupil Diameter (BCPD), respectively (see Implementation of Gaze-Based Measures below). For additional examples of cognitive load measurement via pupil diameter, including examples relevant to HCI see Duchowski et al. [16].

### 2.1 Limitations of pupillometric measures

Given the above review of pupillometric approaches to estimation of cognitive load, which method is most effective and reliable, if any?

**Fig 1:**
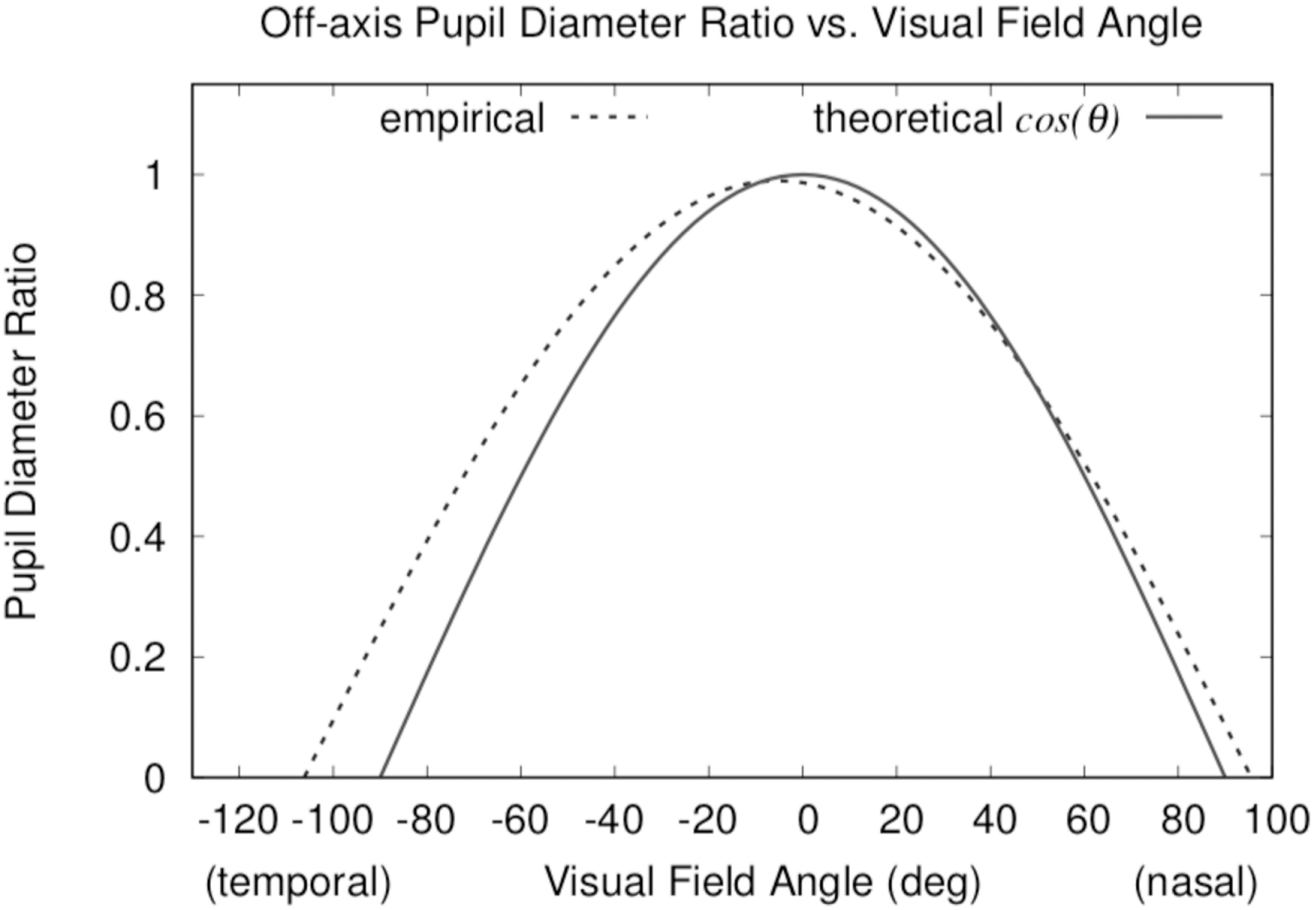
Off-axis pupil diameter ratio. Plot based on model given by Mathur et al. [18]

Persistent problems with eye-tracked measures of pupil diameter, reviewed by Duchowski et al. [16], center on the pupil’s sensitivity to illumination levels and the pupil diameter’s off-axis distortion. This distortion, modeled by Mathur et al. [18] as a function of the cosine of the viewing angle *θ* (in degrees), i.e., *y*(*θ*) = *R*^2^ cos([*θ* + 5.3]/1.121), where *R*^2^ = 0.99, and *y* is the viewing-angle-dependent ratio of the ellipse major and minor axes, is both decentered by a few degrees and flatter by about 12% than the cosine of the viewing angle (see Fig 1).

When using an eye tracker to measure the pupil, effects of illumination and off-axis distortion should be considered. Hayes & Petrov [27] offer a method for compensating for off-axis distortion, while effects of luminance can be handled by considering stimulus nearby the measured point of regard, e.g., as shown by Raiturkar et al. [28]. Duchowski et al. [16] discuss other compensatory approaches as well as techniques based on measuring pupil oscillation, i.e., relative moment-to-moment pupil size.

Here, we consider eye-tracked metrics related to cognitive load based on *positional* eye movement data, derived from measurement of microsaccades.

### 2.2 Microsaccades: Alternative to pupil diameter?

Along with tremor and drift, microsaccades are a component of miniature, involuntary eye movements made during attempted visual fixation [29]. Siegenthaler et al. [19] investigated the influence of task difficulty on microsaccades during the performance of a non-visual, mental arithmetic task with two levels of complexity. They found that microsaccade rates decreased and microsaccade magnitudes increased with increased task difficulty. Microsaccade generation could be affected by working memory performance. In their mental arithmetic study, attention is divided between maintaining fixation and counting tasks, increasing load on working memory. The more difficult the task (i.e., higher working memory load), the more difficult it is to execute the fixation, yielding fewer microsaccades with decreased control over their (e.g., larger) magnitude.

According to Gao et al. [30], non-visual cognitive processing can suppress microsaccade rate, according to the level of task difficulty. When asked to perform easy and difficult arithmetic, participants’ microsaccade rate was modulated at different task phases. In the post-calculation phase, microsaccades remained at double the rate of the calculation phase. Microsaccade rate in the control condition was much greater than during post-calculation.

Dalmaso et al. [31] also reported that working memory load is reflected in microsaccade rate and magnitude. From two experiments, results showed that microsaccadic rate drops in the rebound phase of a high demand task (200-400 ms after onset), compared to an easier task. Results showed a reduction in microsaccadic rate in the high-load condition compared to the low-load condition, consistent with previous findings [19, 30, 32].

Because it is thought that microsaccades and saccades share a common neural generator (the superior colliculus (SC)), Siegenthaler et al. [19] suggest that different levels of task difficulty induce variations in cognitive load, modulating microsaccade parameters via changes in the intensity and shape of the rostral SC activity map. Fluctuations of SC activity at the rostral poles are thought to give rise to microsaccades during fixation.

We should note that Siegenthaler et al. [19] stopped short of positing causality between cognitive workload and microsaccades, suggesting the relation should be probed further, especially in ecologically valid scenarios. However, because the connection was made between task difficulty and microsaccades, the implication that microsaccades may serve as an indicator of cognitive load is tantalizing. They also did not report the effects of task difficulty on eye movement metrics related to pupil diameter, although undoubtedly the eye tracker they used (an EyeLink 1000 sampling at 500 Hz) provided this data. We replicate their experiment comparing and contrasting metrics derived from pupil diameter as captured by an eye tracker.

## 3 Methodology

To compare and contrast microsaccadic indicators with pupillometric measures, we report the results of the eye tracking experiment which followed the experimental design and procedure described by [16] (replicating Siegenthaler et al. [19]). For completeness, below we provide a detailed description of the study methodology, including experimental design, dependent measures and their implementation, procedure, participants, equipment, and statistical analyses.

Present experimental research involved human participants thus it was approved by the SWPS University of Social Sciences and Humanities in Warsaw, Poland Institutional Review Board (IRB). All participants in the study signed informed consent forms in paper.

### 3.1 Experimental design

The experiment followed a 3 × 6 within-subjects design with the following within-subjects fixed factors:

- **Task Difficulty** (Difficult vs. Easy vs. Control). In Difficult and Easy tasks, participants were asked to perform difficult or easy mental calculations, and in the Control task they were not asked to perform any mental calculations at all (see Experimental Procedure below).
- **Time-On-Task** constituted by six blocks of trials within the experimental procedure.

Additionally, each participant’s Working Memory Capacity (WMC) served as a controlled independent variable, measured with the Digit SPAN task (DSPAN) procedure prior to the main experimental task. Both *Forward* and *Backward* assessments of DSPAN [33] were used.

As an indicator of WMC, the length of a correctly recalled numerical sequence (before making two consecutive errors) was used. Each individual’s mean of the two-error maximum length DSPAN from *Backward* and *Forward* assessments was used as a covariant in the statistical analyses.

### 3.2 Dependent measures

We used the following dependent cognitive load measures during the Easy, Difficult and Control tasks.

- We analyzed three microsaccadic metrics. Following Siegenthaler et al. [19], we focused on microsaccade magnitude and rate, and following Di Stasi et al. [34], we analyzed the slopes of the relationship between microsaccadic magnitude and peak velocity.
- We compared two pupillometric metrics, the Intra-Trial (CPD) and the Inter-Trial Change in Pupil Diameter (BCPD).
- Self-assessed cognitive load was also measured. After each block of trials, participants answered a modified NASA Task Load Index (NASA-TLX) questionnaire [35]. The following NASA-TLX items were used: *mental demand*, *physical demand*, *temporal demand*, *performance*, and *effort*. Each item in the questionnaire was evaluated on a Likert-type scale with 1 (“Very Low”) and 21 (“Very High”).

### 3.3 Implementation of gaze-based measures

Prior to implementation of our gaze-based measures, following Engbert and Kliegl [29], eye movement data is first extracted in a pre-processing step to remove data 200 ms before the start of, and 200 ms following the end of a blink, as identified by the eye tracker. Following this pre-processing step, we then compute the inter-trial change in pupil diameter in relation to a base trial, e.g., the intra-trial Change in Pupil Diameter (CPD), Baseline Change in Pupil Diameter (BCPD), and microsaccade magnitude, rate, and peak velocity.

For both the BCPD and CPD pupillary estimates (but for no other measures), we follow Klingner et al. [21] by applying a Butterworth smoothing filter to the raw pupil diameter data prior to computing the metrics based on change in pupil diameter. Butterworth filter parameters were chosen so as to remove high-frequency noise observed in the signal. We take as input signal *x*(*t*) and produce as output its filtered version $\hat{x}\left(t\right)$, where the $\hat{\cdot }$ operator denotes smoothing. We use a 2^*nd*^ degree Butterworth filter with critical frequency set to 1/4 half-cycles per sample, i.e., 1/8^*th*^ the sampling period (the point at which the gain drops to $1/\sqrt{2}$ of the passband). That is, representing the pupil diameter signal as *x*(*t*), the signal is smoothed (to order *s*) by convolving 2*p*+1 inputs *x*_*i*_ with filter $h_{i}^{r,s}$ and 2*q*+1 (previous) outputs ${\hat{x}}_{i}$ with filter $g_{i}^{r,s}$ at midpoint *i* [36]:

$$\begin{array}{c} {\hat{x}}_{n}^{s}\left(t\right)=\frac{1}{\left(\Delta t^{s}\right)}(\sum_{i=-p}^{p}h_{i}^{t,s}x_{n-i}-\sum_{i=-q}^{q}g_{i}^{t,s}{\hat{x}}_{n-i}) \end{array}$$ (1)

where *r* and *s* denote the polynomial fit to the data and its derivative order.

#### 3.3.1 CPD: Intra-trial change in pupil diameter

Using the smoothed pupil diameter signal ${\hat{x}}_{n}^{s}\left(t\right)$ from (1), let $\mu_{T_{b}}$ represent the average smoothed pupil diameter computed over baseline time period *T*_*b*_ as the running mean [37] for *t* ∈ [0, *T*_*b*_] with *k* = 0 incrementing by 1 at each time sample, $\begin{array}{c} \mu_{T_{b}}=k/\left(k+1\right)\mu_{T_{b}}+1/\left(k+1\right){\hat{x}}_{n}^{s}\left(t\right) \end{array}$. The intra-trial change in pupil diameter (CPD) is then computed as the mean difference between the pupil diameter and the average over period *T*_*b*_,

$$\begin{array}{c} \text{CPD}=\frac{k}{k+1}\text{CPD}+\frac{1}{k+1}({\hat{x}}_{n}^{s}\left(t\right)-\mu_{T_{b}}), \end{array}$$ (2)

computed once again as a running mean, for *t* ∈ [0, *T*_*e*_], where *T*_*e*_ is the temporal extent of the CPD estimate. The baseline and extent time periods *T*_*b*_, *T*_*e*_ can be set arbitrarily, e.g., *T*_*b*_ set to the first 2 seconds as is done in the analysis. or depending on how the experiment was set up to include a baseline period (e.g., rest, no induced cognitive load), and *T*_*e*_ set to the entire trial duration or shorter, e.g., 180 seconds, as is done in the analysis below.

#### 3.3.2 BCPD: Inter-trial change in pupil diameter

The inter-trial change in pupil diameter (BCPD) is computed similarly to the CPD as given in (2), with the exception of the baseline average smoothed pupil diameter $\mu_{T_{b}}$ obtained from an entirely different trial, ideally one that is designated as a trial meant not to induce cognitive load. Note that the baseline time period in this case (*T*_*b*_) can extend over the entire trial.

#### 3.3.3 Microsaccade magnitude, rate, peak velocity

Microsaccades can be detected in the raw (unfiltered by the eye tracking software) eye movement signal, **p**_*t*_ = (*x*(*t*), *y*(*t*)), when gaze is fixed on a stationary object, i.e., during a fixation, following fixation detection. Assuming a sequence of raw gaze points identified within a fixation, we adapt a version of Engbert and Kliegl’s [29] algorithm for the detection of microsaccades.

The algorithm proceeds in three steps. First, we transform the time series of gaze positions to velocities via

$$\begin{array}{ccc} {\dot{x}}_{n} & = & \frac{x_{n+2}+x_{n+1}-x_{n-1}-x_{n-2}}{6\Delta t}, \end{array}$$ (3)

but do so separably for *x*(*t*) and *y*(*t*). Eq (3) represents a moving average of velocities over 5 data samples. As Engbert and Kliegl [29] note, due to the random orientations of the velocity vectors during fixation, the resulting mean value is effectively zero. Microsaccades, being ballistic movements creating small linear sequences embedded in the rather erratic fixation trajectory induced by small drifts, can therefore be identified by their velocities, which are clearly separated from the kernel of the distribution as “outliers” in velocity space.

Second, computation of velocity thresholds for the detection algorithm is based on the median of the velocity time series to protect the analysis from noise. A multiple of the standard deviation of the velocity distribution is used as the detection threshold [38],

$$\sigma_{x}=\sqrt{\left\langle {\dot{x}}^{2}\right\rangle -{\left\langle \dot{x}\right\rangle }^{2}},\;\;\;\sigma_{y}=\sqrt{\left\langle {\dot{y}}^{2}\right\rangle -{\left\langle \dot{y}\right\rangle }^{2}}$$

where 〈⋅〉 denotes the median estimator. Detection thresholds are computed independently for horizontal *η*_*x*_ and vertical *η*_*y*_ components and separately for each trial, relative to the noise level, i.e., *η*_*x*_ = λ*σ*_*x*_, *η*_*y*_ = λ*σ*_*y*_. Like Engbert and Kliegl [29], we used λ = 6 in all computations and we assume a minimal microsaccade duration of 6 ms (three data samples at 500 Hz). Mergenthaler [39] discusses how the choice of λ substantially affects the number of detected microsaccades. As λ increases, the number of detected microsaccades decreases. Following Engbert [38], as a necessary condition for a microsaccade, we require $\dot{x}$ and $\dot{y}$ fulfill the criterion ${\left({\dot{x}}_{n}/\eta_{x}\right)}^{2}+{\left({\dot{y}}_{n}/\eta_{y}\right)}^{2}>1$.

Third, Engbert and Kliegl [29] focus on binocular microsaccades, defined as microsaccades occurring in left and right eyes with a temporal overlap. They exploit binocular information by applying a temporal overlap criterion: if a microsaccade in the right eye starting at time *r*_1_ is found that ends at time *r*_2_, and a microsaccade in the left eye begins at time *l*_1_ and ends at time *l*_2_, then the criterion for temporal overlap is implemented by the conditions *r*_2_ > *l*_1_ and *r*_1_ < *l*_2_. We omit this step to facilitate working with a single (unfiltered) value for gaze point velocity estimation within a fixation. We follow Duchowski et al. [40] procedure and average both left and right gaze points into a single point as would be looked at by a cyclopean eye, i.e., (*x*(*t*), *y*(*t*)) = ([*x*_*l*_(*t*) + *x*_*r*_(*t*)]/2, [*y*_*l*_(*t*) + *y*_*r*_(*t*)]/2). However, unlike Duchowski et al. [40] we do not implement heuristic data mirroring, which may make our approach susceptible to eye tracker noise, especially in cases when only one of the left or right data points is available. Heuristic data mirroring may counteract this problem.

**Fig 2:**
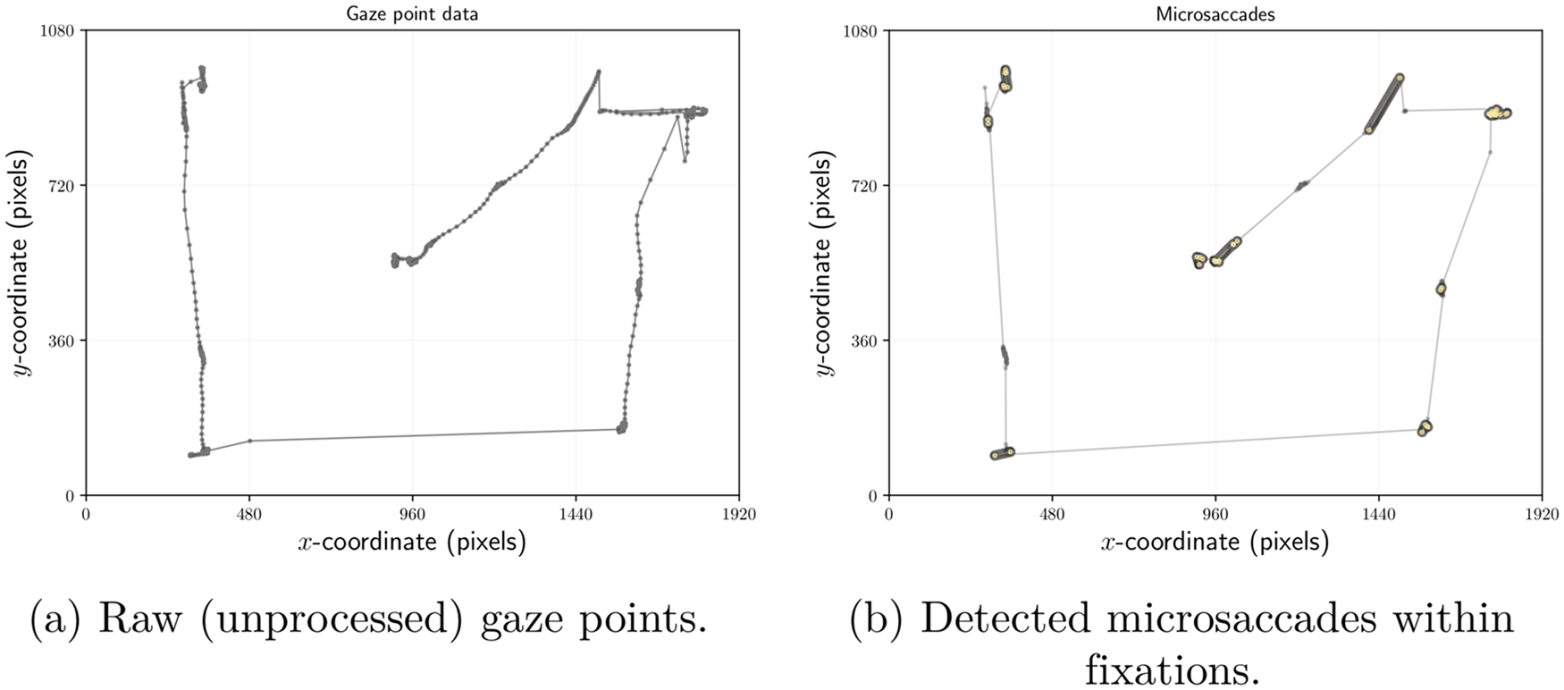
Illustration of microsaccade detection in a single participant’s gaze recording during calibration. The small dots in (a) show gaze points prior to processing, i.e., detection of fixations or of microsaccades. Following processing, microsaccades in (b) are highlighted by larger, darker dots and thicker connecting segments. Notice that all small, raw gaze points that were not part of a fixation have now been removed. Thus any remaining small dots are members of fixation clusters that may or may not contain microsaccades. The point of this illustration is to show that microsaccades are detectable at locations far beyond the central fixation point. In the replication of Siegenthaler et al.’s [19] study, however, the participant’s gaze was held fixed at a central fixation point, hence the microsaccades depicted here during calibration were not used in the analysis of the experiment.

Note that Engbert and Kliegl [29] assume a stationary eye movement signal, i.e., when fixating an object, e.g., performing a task where gaze is meant to be held steady e.g., see Siegenthaler et al. [19]. This assumption allows processing of the eye movement signal stream in its entirety, with the distinction between saccades and microsaccades made by thresholding on saccade amplitude. However, doing so precludes the grouping of raw (unprocessed) eye movement data within fixations. Moreover, because the entirety of the eye movement recording (scanpath) is needed, the approach also precludes real-time applications. With future real-time HCI applications in mind, we adapted their algorithm to the general case of a non-stationary eye movement signal, by first detecting fixations following Nyström and Holmqvist [41], and by using the Savitzky and Golay [42] filter for velocity-based (I-VT [43]) event detection. The Savitzky-Golay filter fits a polynomial curve of order *n* via least squares minimization prior to calculation of the curve’s *s*^*th*^ derivative e.g., 1^*st*^ derivative (*s* = 1) for velocity estimation [44]. We used a 3^*rd*^ degree Savitzky-Golay filter of width 3 with velocity threshold of 100°/s, tuned to the sampling rate of our eye tracker, see Fig 2.

Because the Savitzky-Golay filter is fairly short, it can be used in real-time applications. Moreover, because our adaptation functions within data points identified within fixations, we can (eventually) test off-axis compensation algorithms as we need not rely on the assumption of stationarity.

### 3.4 Experimental procedure

Participants first signed a consent form, provided basic demographic information, and then completed the DSPAN assessment. The DSPAN was completed on a dedicated laptop computer. The procedure consisted of 14 trials. In each trial participants were presented with a random number (starting with a 3 digit number) viewed for 1 second. Their task was to recall this number in the same (*Forward* assessment) or reverse order (*Backward* assessment). Given a correct response, the digit sequence was extended by 1 digit in the next trial. Given an incorrect response, the length of the next sequence was kept the same. The order of *Forward* and *Backward* assessment blocks was randomized.

**Fig 3:**
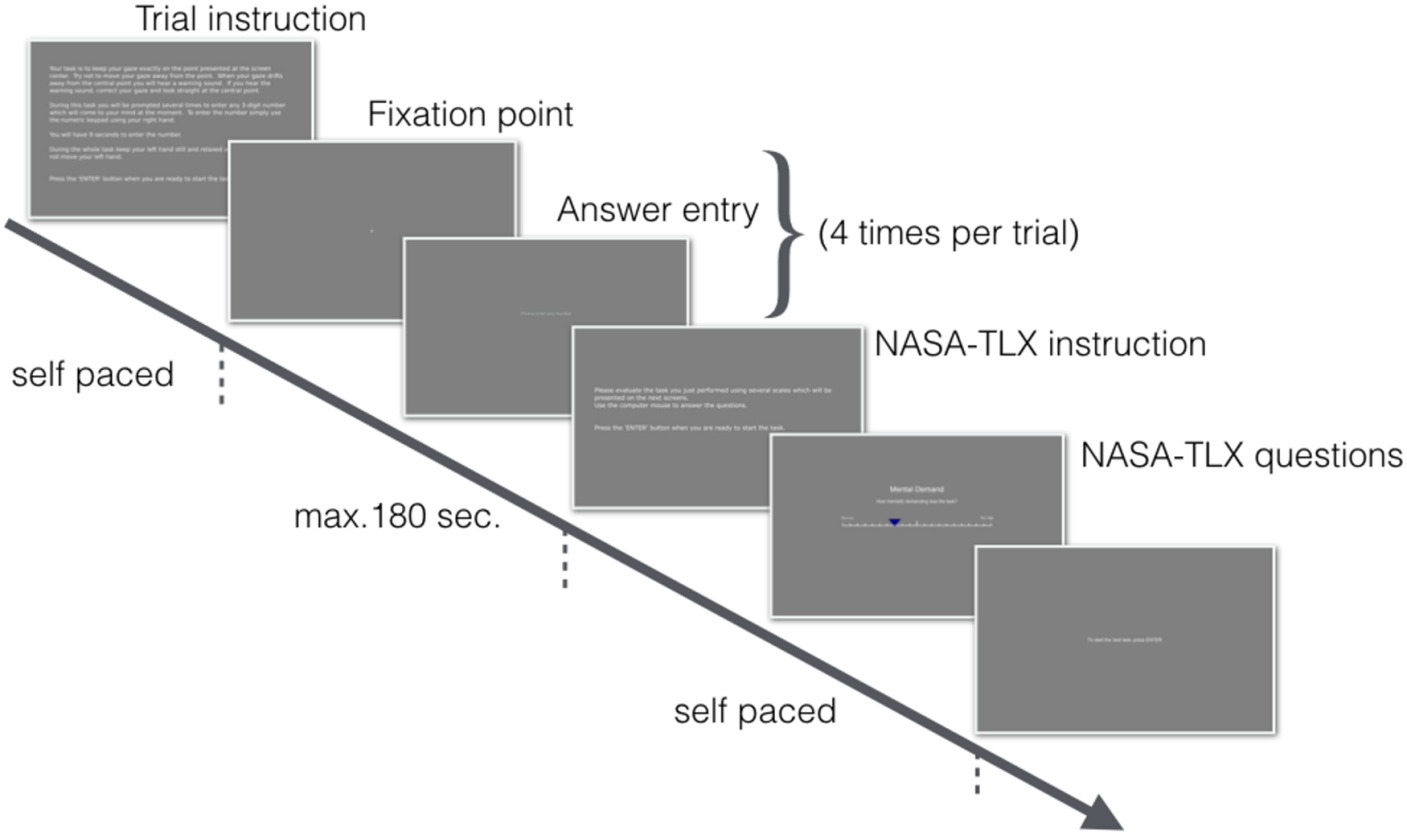
Time course of each experimental trial. Note that because we wanted to present the experimental procedure as accurately as possible the fixation point and text entry text are hardly visible on this figure due to its size. In the actual experimental settings both were clearly visible for participants on the computer screen. The actual fixation point was defined as a circle with radius of 5 pixels and color defined as [0.2,0.2,0.2] in the RGB color scale. That color can be described as an increment of 0.2 above the gray background. Note that each value in the RGB space in PsychoPy is defined by the range between -1 and 1. The same color was applied to the text on the “Answer entry” screen.

As the final step of the procedure, participants completed the main task during which their eyes were tracked following a 5-point calibration (see Fig 3). The main task consisted of three types of mental computation trials: Difficult, Easy, and Control. Trials were grouped into 6 blocks, giving 18 trials in total. Each block started with the Control trial, followed by the Easy and Difficult trials in counterbalanced order. The order of block of trials was randomized. Between each block, participants were offered to take a break lasting from 2 to 5 minutes.

During each trial (Difficult, Easy and Control) participants were asked to keep their gaze at a central fixation point. Deviation of the gaze > 3° from the fixations point was penalized by an unpleasant warning sound. Each trial started with the presentation of a randomly chosen number from the sets of {1375, 8489, 5901, 5321, 4819, 1817} for Difficult trials and {363, 385, 143, 657, 935, 141} for Easy trials. In Difficult trials, participants were asked to mentally count backwards in steps of 17 as fast and accurately as possible while in Easy trials they were asked to count forward in steps of 2 starting from the initial number. In both types of trials participants were asked 4 times per trial to enter their current calculation and continue counting. Prompts appeared at random times during the trial with a minimum gap between prompts set to 15 seconds and a maximum gap time set to 80 seconds. A limit of 9 seconds was given for entering their current calculation. In the Control trials participants were not asked to perform any mental calculations. However, they were asked to enter any 3-digit number that came to mind when prompted. In each condition the trial duration was set to a maximum of 3 minutes. After finishing each trial participants completed the NASA-TLX questionnaire by estimating their level of cognitive demand in each task.

### 3.5 Experimental sample

Seventeen psychology students volunteered for the study. Data of four participants were discarded due to technical problems (mainly with the eye tracker calibration) or misunderstanding of the task. The final sample consisted of *N* = 13 participants, 7 males and 6 females aged between 20 and 40 years old (*M* = 29.77; *SD* = 7.15).

### 3.6 Experimental equipment

Eye movements were recorded binocularly by an SR Research EyeLink 1000 eye tracker (500 Hz sampling rate). During the recording each participant’s head was stabilized with a chin rest. The accuracy of the eye tracker reported by SR Research is 0.25°–0.5° visual angle on average, with microsaccade resolution of 0.05°. However, Van Der Geest [45] reported lower horizontal × vertical precision (0.98° × 1.05° visual angle).

The stimuli were presented on a computer screen with 1920 × 1080 resolution. The main experiment procedure was created in PsychoPy [46]. Participants responded using a numerical keyboard. The DSPAN test was performed using Millisecond Inc.’s Inquisit 4 Lab software.

The experimental room had no windows and ambient light was controlled for each of the participants (520 lux). Luminance of the computer screen during the experimental task was measured at 120-130 lux.

## 4 Results

A useful measure of cognitive load should be sensitive to both between-task and within-task variability as well as between-subjects differences [47]. First we report internal validity (reliability) of questionnaire responses and pupillary and microsaccadic measures of cognitive load. Second we report external validity (sensitivity) of the measures reflected by their ability to distinguish between Task Difficulty within sequential blocks of trials (i.e., Time-On-Task). We also report on each measure’s ability to distinguish between high and low cognitive load as a between-subjects factor. Finally, we give a direct comparison of the measures by testing a multinomial logistic regression (MLR) model for discriminating between task difficulty.

### 4.1 Statistical analyses

The internal validity of all measures was assessed with Cronbach’s *α*. In order to evaluate the influence of Task Difficulty and Time-On-Task on dependent measures, two-way (3 × 6) within-subjects Analyses of Covariance (ANCOVAs) were used, where Task Difficulty (Control vs. Easy vs. Difficult) and Time-On-Task (block of trials from 1 to 6) served as independent factors. The analyses of covariance were followed by pairwise comparisons with HSD Tukey’s correction when needed.

Working memory capacity can be considered as a moderator between eye-related measures and cognitive load, e.g., Granholm et al. [48] showed that pupillary response increases with increasing task demand until cognitive resources are exceeded, at which point pupillary response then begins to decline. Past work has also reported significant relationships between working memory capacity and performance of various complex cognitive tasks e.g., reading comprehension, and reasoning [49, 50]. One may expect that people with high working memory capacity should experience lower cognitive load in difficult tasks than individuals with low working memory capacity. However, to our best knowledge no direct relationship between working memory capacity and fixational eye movements has been described in the literature. Thus, we decided to include working memory capacity as a covariant in the statistical analyses of all dependent measure sensitivities.

We used parametric ANCOVA statistical tests despite the fact that all microsaccadic and pupillary measures showed skewed distributions deviating from normality. ANCOVA allowed us to run full design analyses and is considered relatively robust to violation of the normality assumption, e.g., see Schmider et al. [51] or Lix et al. [52]. All ANCOVA results are reported with generalized main effect size (*η*^2^). For direct comparison of eye movement measures multinomial logistic regression analyses were used.

Note that prior to statistical analyses, microsaccades were additionally filtered using thresholds of maximum duration (40 ms) and maximum magnitude (2 visual degrees); for review of microsaccades filtering see Otero-Millan et al. [53] and Martinez-Conde et al. [54].

All statistical analyses were conducted in R [55].

### 4.2 Reliability of measures

Reliability (internal consistency) of microsaccadic and pupillary responses are estimated by Cronbach’s *α* and compared to self-reported (NASA-TLX questionnaire) measures (see Table 1). Both eye movement and self-reported measures show very good reliability, in most cases (*α* ≥ 0.80) acceptable [56]. Excellent reliability (*α* > 0.90) was obtained for microsaccade magnitude, microsaccade rate, and Inter-Trial Change in Pupil Diameter, overall, and for each task. The lowest Cronbach’s *α* was found for peak velocity and microsaccade magnitude slopes in the Difficult tasks.

**Table 1:** Internal consistency of cognitive load measures in response to task difficulty. The table presents Cronbach’s *α* overall and within each task. The reliability coefficient could not be calculated for the BCPD measure in the Control tasks as it was treated as the baseline. Note the meaning of abbreviations in the table: **MM** (Microsaccades Magnitude), **MR** (Microsaccade Rate), **PV-M** (Microsaccade Peak Velocity—Magnitude), **CPD** (Intra-Trial Change in Pupil Dilation), **BCPD** (Inter-Trial Change in Pupil Dilation).

Variable	Overall	Task		
		Control	Easy	Difficult
MM (deg)	0.96	0.97	0.95	0.97
MR (Hz)	0.96	0.96	0.96	0.96
MPV-M intercept	0.82	0.85	0.79	0.83
MPV-M slope	0.80	0.88	0.78	0.57
CPD	0.86	0.84	0.94	0.73
BCPD	0.95	—	0.92	0.95
NASA-TLX	0.88	0.83	0.87	0.77

### 4.3 Experimental manipulation check

**Fig 4:**
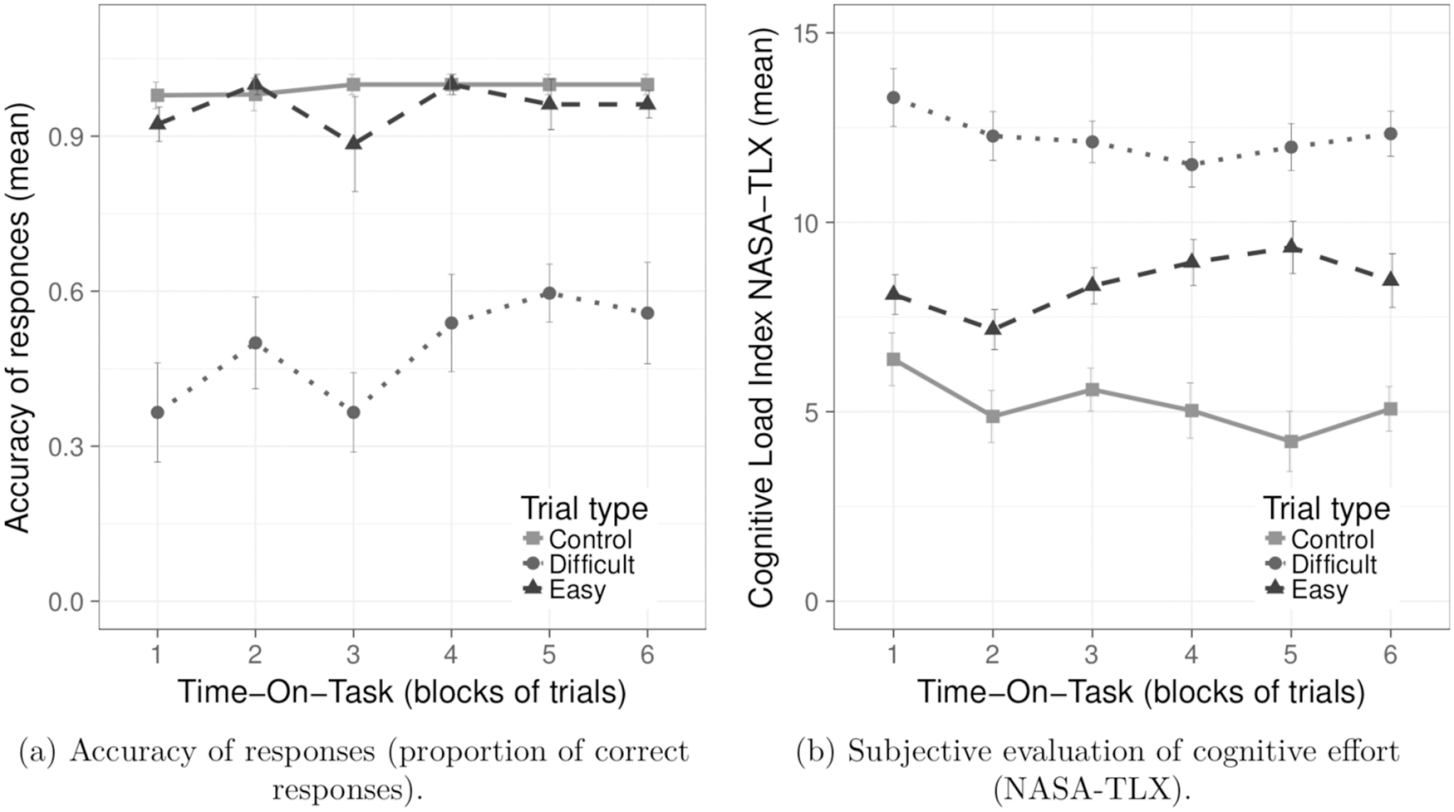
Manipulation check and questionnaire data. Results on response accuracy and subjective evaluation of effort. Means are plotted on trial type and Time-On-Task. The error bars represent ± 1 SE for the means.

To gauge the effect of task difficulty on participants’ performance, we analyzed task response accuracy (proportion of correct responses) and self-reported cognitive load (raw TLX index), see Fig 4.

The accuracy of the counting task in Difficult trials was checked by examining divisibility by 17 of the difference between the participant’s current response and the previously entered or starting number. In the Easy trials accurate answers were defined as any positive even difference between starting number or previously entered number and the current response. In Control trials accurate answers were any 3-digit numbers entered by the participants. The performance criterion for Difficult trials was a minimum of 4 out of 24 correct answers. Based on this criterion, the data of one subject, who only scored 3 correct answers in all of the Difficult tasks was removed from further analyses. Note that no data were discarded from the Easy and Control trials.

As expected, ANCOVA of performance accuracy (response accuracy proportion) revealed a main effect of task difficulty, *F*(2, 20) = 54.43, *p* < 0.001, *η*^2^ = 0.58, see Fig 4(a). Participants in the Difficult tasks produced significantly fewer correct answers than in the Easy and Control tasks (*p* < 0.001), see Table 2. The analyses revealed also a significant interaction effect (but relatively weak in terms of its effect size) between WMC and Time-On-Task *F*(2, 20) = 4.15, *p* < 0.05, *η*^2^ = 0.09.

**Table 2:** Dependent variables’ descriptive statistics overall and for each task: Means and standard errors (SE). Note the meaning of abbreviations in the table: **CA** (Correct Answers), **MM** (Microsaccades Magnitude), **MR** (Microsaccade Rate), **MPV-M** (Microsaccade Peak Velocity—Magnitude), **CPD** (Intra-Trial Change in Pupil Dilation), **BCPD** (Inter-Trial Change in Pupil Dilation).

Variable	Overall	Task		
		Control	Easy	Difficult
CA (prop.)	0.81 (0.02)	0.99 (0.01)	0.97 (0.01)	0.49 (0.04)
NASA-TLX	8.61 (0.32)	5.19 (0.38)	8.39 (0.50)	12.26 (0.46)
MM (deg)	0.43 (0.01)	0.39 (0.01)	0.43 (0.01)	0.46 (0.01)
MR (Hz)	1.36 (0.03)	1.26 (0.05)	1.43 (0.06)	1.41 (0.06)
PV-M intercept	-12.82 (0.41)	-12.88 (0.70)	-13.69 (0.69)	-11.90 (0.70)
PV-M slope	216.59 (1.04)	216.17 (2.26)	216.26 (1.62)	217.35 (1.43)
CPD	-51.02 (4.50)	-59.25 (7.35)	-66.79 (7.35)	-27.03 (8.02)
BCPD	45.04 (5.62)	— (—)	17.37 (7.00)	72.71 (7.64)

Results of the self-reported questionnaire responses are in line with those of performance accuracy. A two-way ANCOVA of the Raw NASA TLX scale showed a significant main effect of task difficulty, *F*(1.31, 14.46) = 46.09, *p* < 0.001, *η*^2^ = 0.38, see Fig 4(b). Participants reported significantly higher cognitive load during the Difficult tasks than during the Easy and Control tasks (*p* < 0.001). Pairwise comparisons showed that the difference between the Easy and Control task was statistically significant (*p* < 0.01), see Table 2.

ANCOVA of the Raw NASA TLX score also revealed a statistically significant interaction effect of Task Difficulty and Time-On-Task, *F*(4.64, 51.06) = 2.91, *p* < 0.05, *η*^2^ = 0.02, see Fig 4(b). Pairwise comparisons of means showed that in all blocks of trials the difference between Easy and Control tasks in the TLX score was significant (*p* < 0.001) in favor of the Easy tasks in all but the first block of trials where the difference was not significant (*p* > 0.1). We observed significantly greater self-reported cognitive load following Difficult tasks in comparison to the Easy and Control tasks (*p* < 0.001).

### 4.4 Microsaccade main sequence

**Fig 5:**
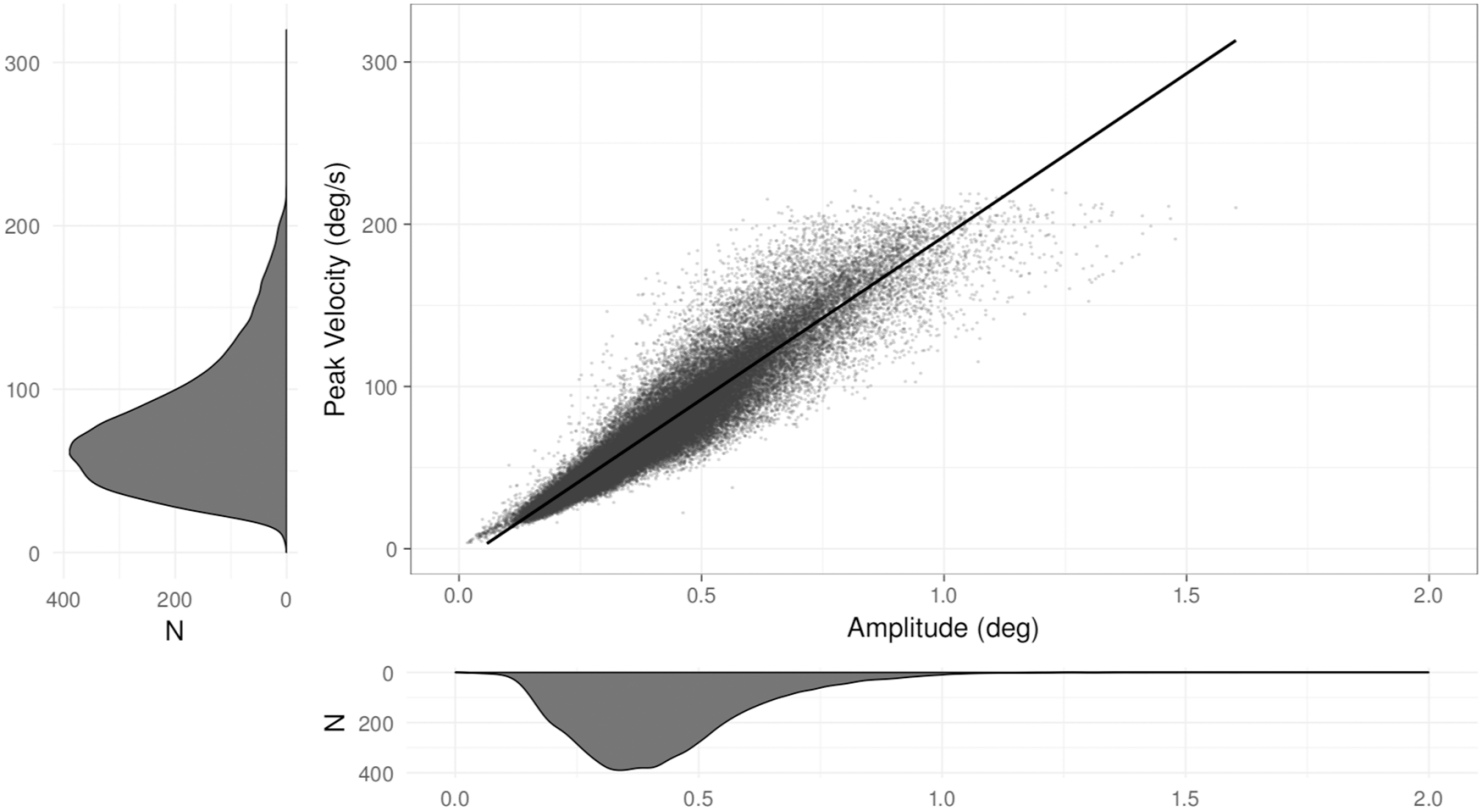
Microsaccadic peak velocity vs. magnitude (main sequence). Main panel: the scatter plot represents detected microsaccades with amplitude indicated on the abscissa and peak velocity on the ordinate. The line drawn through is a linear fit through the scatter plot. Bottom and side panels: microsaccade amplitude and peak velocity distributions (*N* = 13 subjects).

We expected a linear relation between microsaccade peak velocity and magnitude, i.e., the (micro-)saccadic *main sequence* [19, 57, 58]. Indeed, a linear regression on microsaccade magnitude and peak velocity was significant, *F*(1, 71281) = 424800, *p* < 0.001 with *R*^2^ = 0.856, see Fig 5.

Tests of normality showed that the frequency distributions of all microsaccadic measures deviated from normality. Specifically, one-sample Kolmogorov-Smirnov tests of normality were statistically significant (showing deviation from normality), for each of the following distributions: microsaccade magnitude (*D* = 0.59, *p* < 0.001), microsaccade rate (*D* = 0.70, *p* < 0.001), microsaccade peak velocity (*D* = 1, *p* < 0.001), slopes (*D* = 1, *p* < 0.001) and intercepts (*D* = 0.93, *p* < 0.001), see Fig 5.

Our normality distribution tests fail to corroborate results of Siegenthaler et al. [19] although their claims of normality may seem odd given similar skews in their distribution plots.

### 4.5 Microsaccade response to task difficulty

Microsaccadic reaction to task difficulty was evaluated using the following dependent measures in a series of two-way ANCOVAs: microsaccade magnitude, microsaccade rate, and slope and intercept of the relation between microsaccade peak velocity and magnitude (for the last two, see Siegenthaler et al. [19] and Di Stasi et al. [34]).

#### 4.5.1 Microsaccade magnitude

**Fig 6:**
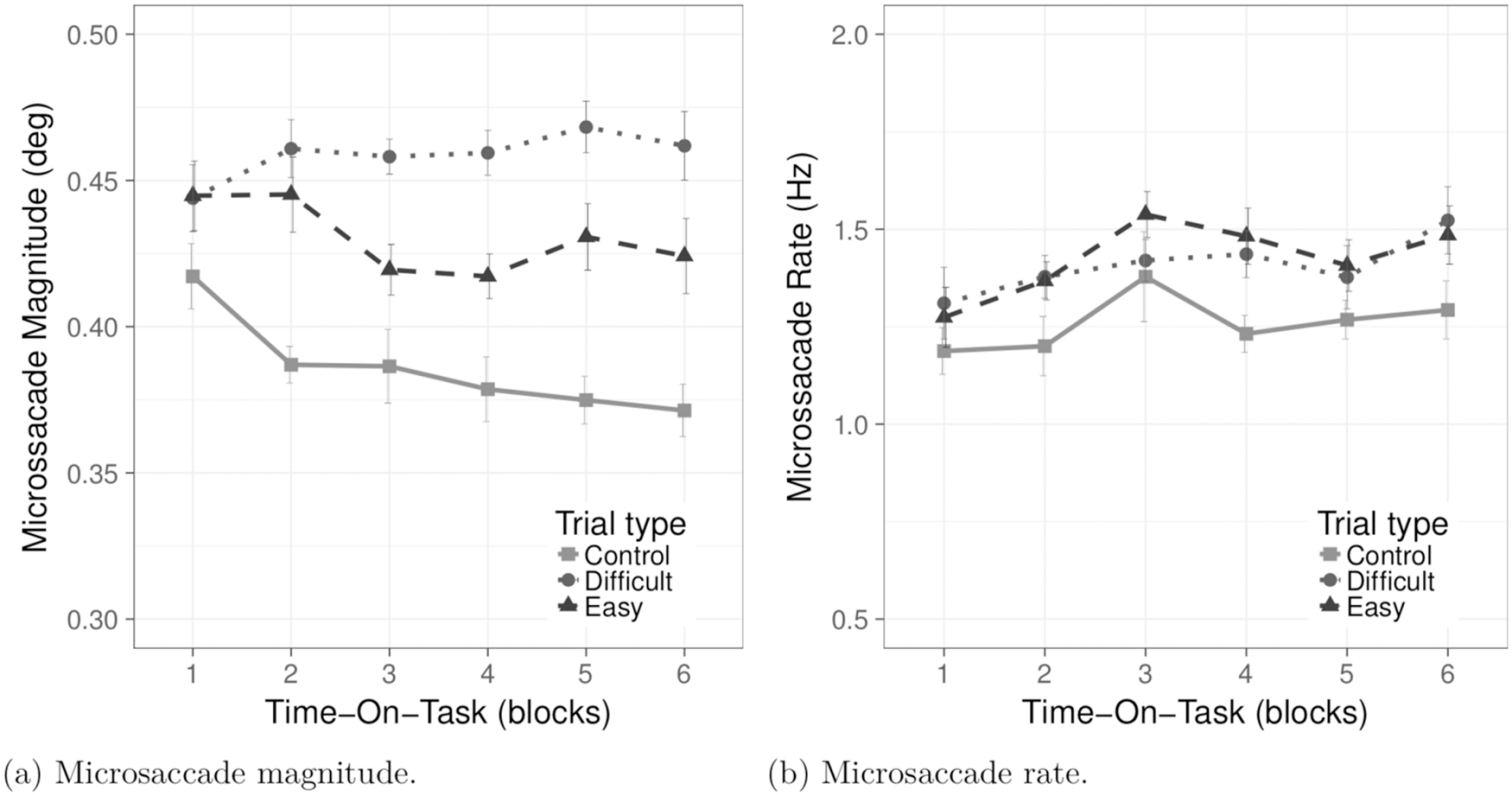
Microsaccade magnitude, rate in response to task difficulty and Time-On-Task. Means are plotted for trial type versus Time-On-Task. Error bars represents ± 1 SE for the means.

We expected to see greater microsaccade magnitude when performing the Difficult tasks in comparison to the Control and Easy tasks. In line with this hypothesis, ANCOVA of microsaccade magnitude revealed a main effect of Task Difficulty, *F*(1.79, 19.74) = 32.39, *p* < 0.001, *η*^2^ = 0.17. Pairwise comparisons with Tukey correction showed a statistically significant difference in microsaccade magnitude between all tasks (*p* < 0.01). Microsaccade magnitude was highest during the Difficult tasks, lower during the Easy tasks, and lowest during the Control tasks (see Fig 6(a) and Table 2 for descriptive statistics).

ANCOVA also revealed a statistically significant interaction effect of Task Difficulty and Time-On-Task, showing that microsaccade magnitude depends on Task Difficulty as well as on Time-On-Task. Differences between the Easy and Difficult tasks increase during the course of the trials, *F*(4.53, 49.88) = 3.38, *p* < 0.01, *η*^2^ = 0.02, see Fig 6(a). Post-hoc analyses showed that the difference between Task Difficulty was not significant in the 1^*st*^ block of trials (*p* > 0.05). In the 2^*nd*^ block of trials, microsaccade magnitude decreased significantly (*p* < 0.001) in the Control tasks compared to the Easy and Difficult tasks, although microsaccade magnitude between the latter two did not differ significantly (*p* > 0.1). From the 3^*rd*^ block of trials onwards, microsaccade magnitude was significantly greater in the Difficult tasks compared to the Control and Easy tasks (*p* < 0.02). The difference between the Control and Easy tasks became significant from the 5^*th*^ block of trials onwards (*p* < 0.001).

#### 4.5.2 Microsaccade rate

Contrary to expectations, only a marginally significant main effect of task was found on microsaccade rate, *F*(1.84, 20.27) = 3.14, *p* = 0.06, see Fig 6(b). Pairwise comparisons revealed a marginally significant difference (*p* = 0.07) between the Easy and Control tasks, with microsaccade rate greater during the Easy tasks than during the Control tasks. For full descriptive statistics see Table 2.

#### 4.5.3 Microsaccade peak velocity and magnitude

Also contrary to expectations, ANCOVA of the relation (slope) between microsaccade peak velocity and magnitude showed no statistically significant effect of task, *F*(1.59, 17.49) < 1. No other effects, main or interaction, reached significance.

Finally, no statistically significant effects were found for intercepts of the relation between microsaccade peak velocity and magnitude, *F*(1.63, 17.91) = 1.20, *p* > 0.1. For descriptive statistics see Table 2.

### 4.6 Pupillary response to task difficulty

Pupillary response to task difficulty was tested by two independent indicators, inter-trial change in pupil diameter (BCPD), and intra-trial change of pupil diameter (CPD). According to the literature, both measures should indicate differences in cognitive load, distinguishing between Difficult, Easy, and Control tasks. To test this hypothesis both CPD and BCPD were used as dependent measures in two independent within-subjects ANCOVAs with Task Difficulty and Time-On-Task as fixed factors. Working memory capacity was used as a covariate.

As with microsaccadic measures, pupillary measures also failed the test for distribution normality. We checked distribution normality of both CPD and BCPD with a one-sample Kolmogorov-Smirnov test, and both deviated significantly: for CPD, *D* = 0.79, *p* < 0.001, and for BCPD, *D* = 0.74, *p* < 0.001.

#### 4.6.1 Intra-trial change in pupil diameter

**Fig 7:**
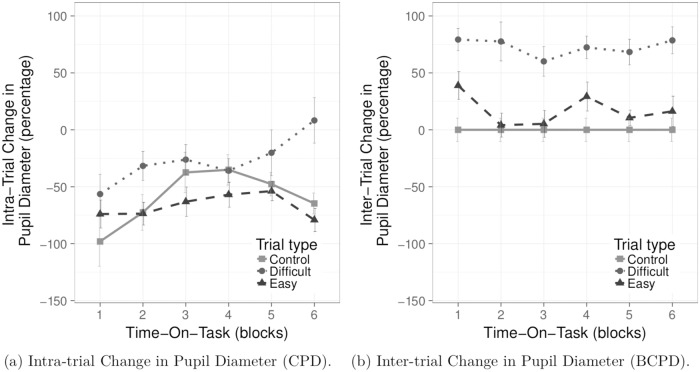
Pupil response to task difficulty and Time-On-Task. Plots show mean change in pupil diameter versus Time-On-Task; error bars represent ± 1 SE.

CPD shows pupillary constriction during the course of the trial. However, as expected, pupil diameter tends to remain relatively more dilated during the Difficult task than during both Easy and Control tasks. ANCOVA of CPD revealed a main effect of task, *F*(1.26, 13.89) = 6.44, *p* < 0.05, *η*^2^ = 0.07, see Fig 7(a). Post-hoc analyses showed that CPD differed significantly in response to the Difficult tasks compared to the Easy tasks (*p* < 0.02). The difference between the Difficult and the Control tasks failed to reach significance (*p* = 0.071); the difference between the Easy and the Control tasks was not statistically significant (*p* > 0.1). In line with hypotheses, pupil diameter tends to dilate during the Difficult task compared to both the Control and the Easy tasks, see Table 2. The analysis showed no other statistically significant effects.

#### 4.6.2 Inter-trial change in pupil diameter

As expected, BCPD shows increased pupillary dilation during the Difficult tasks compared to the Easy tasks. ANCOVA of BCPD excluded the Control tasks as these tasks constituted the baseline (producing a constant nil inter-trial difference). Analysis revealed a statistically significant main effect of task, *F*(1, 11) = 25.15, *p* < 0.001, *η*^2^ = 0.16, see Fig 7(b). For both Easy and Difficult tasks, pupil diameter dilated from the baseline (the Control task) but for the Difficult tasks the difference was significantly greater compared to the Easy tasks, see Table 2.

### 4.7 Pupillary and microsaccadic effect sizes

Analyses of covariance (ANCOVA) showed that microsaccade magnitude, intra-trial change in pupil diameter (CPD), and inter-trial change in pupil diameter (BCPD) produced mean indicators that differed significantly among the Difficult, Easy, and Control tasks. Comparison of task effect sizes showed that task difficulty explained the highest portion of variance for microsaccade magnitude (*η*^2^ = 0.17) and BCPD (*η*^2^ = 0.16) while for CPD the effect was noticeably smaller (*η*^2^ = 0.07).

### 4.8 Multinomial logistic regression

Direct comparison of the sensitivity of pupillary and microsaccadic responses to task difficulty was performed by multinomial logistic regression (MLR) modeling. Multinomial logistic regression is a form of linear regression analysis conducted for nominal dependent variables that exceed two levels. It is often considered as an alternative to discriminant analysis. In the tested model, Task Difficulty was used as the dependent variable and the Control task was used as the reference. Microsaccadic and pupillary measures (microsaccade magnitude, microsaccade rate, peak velocity/magnitude slope, CPD, and BCPD) were used as input to the model as predictors. Prior to model input, all predictive measures were standardized, meaning that their scales were mapped to *z*-scores, according to statistical standardization, $z_{i}=\left(x_{i}-\bar{x}\right)/\sigma_{x}$ where $\bar{x}$ and *σ*_*x*_ are mean and standard deviation, respectively.

The fit MLR model gauges which of the pupillary and microsaccadic measures best distinguish the Easy and Difficult tasks from the Control tasks (the Control tasks were treated as a reference in the model). Both BCPD and microsaccade magnitude significantly predict log odds of performing either of the Easy and Difficult tasks in reference to the Control task. Moreover, CPD and microsaccade rate significantly predict log odds of performing only the Easy task in reference to the Control, see Table 3.

**Table 3:** Regression coefficients (*β*) and standard errors (in parentheses) of two multinomial logistic regression analyses. In the first (Difficult vs. Control) and the second (Easy vs. Control) analysis, Control task served as reference. The intercept coefficient for the Difficult task was not statistically significant, *β*_0_ = 0.39, SE = 0.26, *z* = 1.49, *p* > 0.05, but the intercept was significantly different from zero for the Easy task, *β*_0_ = 0.65, SE = 0.25, *z* = 2.63, *p* < 0.01. Statistical significance of all *β* coefficients was checked with a Wald z-test; p-values are marked with asterisks (**p < 0.001, *p < 0.05).

Variable	Difficult vs. Control	Easy vs. Control
MS magnitude	1.20** (0.25)	0.82** (0.21)
MS rate	0.25 (0.21)	0.38* (0.18)
MS slopes	0.42 (0.22)	0.26 (0.19)
Intra-Trial CPD	−0.09 (0.25)	−0.58* (0.21)
Inter-Trial BCPD	2.47** (0.42)	1.43** (0.39)

Closer investigation of MLR model coefficients in Table 3 shows that one standard deviation increase in BCPD significantly increases the log odds of performing the Difficult task vs. the Control task (*β* = 2.47, SE = 0.42, *z* = 5.90, *p* < 0.001). Similarly, an increase of microsaccade magnitude by one standard deviation significantly increases the log odds of performing the Difficult task (*β* = 1.20, SE = 0.25, *z* = 4.81, *p* < 0.001). The model also shows that BCPD (*β* = 1.43, SE = 0.39, *z* = 3.68, *p* < 0.001) and microsaccade magnitude (*β* = 0.82, SE = 0.21, *z* = 3.82, *p* < 0.001) significantly increases the log odds of performing the Easy tasks vs. the Control task.

An increase of 1 *SD* of microsaccade rate significantly increases the log odds of performing Easy task (*β* = 0.38, SE = 0.18, *z* = 2.07, *p* < 0.05). Surprisingly, an increase of microsaccade rate did not significantly predict log odds of performing the Difficult task (*β* = 0.25, SE = 0.21, *z* = 1.15, *p* > 0.1).

Notice that both CPD and microsaccade rate predict the log odds of performing only the Easy tasks in comparison to the Control and the Difficult tasks. An increase of one standard deviation in CPD decreases the log odds of performing the Easy task (*β* = −0.51, SE = 0.21, *z* = 2.40, *p* < 0.02).

## 5 Summary and discussion

Task-evoked microsaccadic and pupillary measures were compared in response to elicited mental tasks at three levels of difficulty. Participants were asked to perform difficult and easy mental calculations and to perform no specific task at all. We believe differing levels of task difficulty lead to differing levels of cognitive load. We therefore expected both types of eye-related (microsaccadic and pupillary) measures to reflect changes in response to differing cognitive demands.

Task difficulty was evaluated with response accuracy. Participants gave fewer correct responses to Difficult tasks compared to the Easy and Control tasks. Participants also consistently self-reported greater cognitive load during performance of the Difficult task than the Easy (addition) and Control tasks.

Analyses of internal consistency (reliability) of eye-related measures revealed that microsaccade magnitude, microsaccade rate and inter-trial change in pupil diameter showed high reliability. Remaining measures, including responses to the NASA-TLX questionnaire showed good (acceptable) reliability.

Analyses of external consistency (sensitivity) of eye-related measures showed variability of microsaccadic and pupillary responses, i.e., in their ability to capture differences in cognitive responses to task difficulty. Below we summarize the sensitivity of both pupil-based and microsaccadic measures.

Microsaccade magnitude was found to be sensitive to task difficulty, observed at significantly higher levels during performance of difficult tasks than during performance of the easy or control tasks. This is consistent with previous findings, in particular those of Siegenthaler et al. [19] and Di Stasi [34]. Furthermore, the difference between tasks appeared to become increasingly salient during the time course of the experiment, i.e., microsaccade magnitude increased during performance of the difficult task while it decreased during performance of the easy and control tasks. Task difficulty explained 16% of microsaccade magnitude variance.

Contrary to expectations, neither microsaccade rate nor the relationship between microsaccade peak velocity and magnitude was sensitive to task difficulty or to Time-On-Task. Although Siegenthaler et al. [19] reported a decrease in microsaccadic rate during difficult tasks, we cannot corroborate their findings. We obtained an only marginally significant difference in microsaccade rate between the Easy and Control tasks (the rate was higher in Easy tasks than in Control ones). As Siegenthaler et al. [19] noted, microsaccade rates have produced varied (inconsistent) results in response to varied task difficulty, e.g., decreased rates during high-difficulty visual tasks, or increased rates with task difficulty (albeit given different task demands).

Among the pupillary response measures, both intra- and inter-trial change in pupil diameter showed significant sensitivity to task difficulty. However, they differed in their interpretive clarity and in their capacity for explanation of variance in the data. Inter-trial BCPD offered a much clearer interpretation, with mean differences reflecting response to task difficulty. It is also worth noting that the two pupillary response measures differed in terms of their power. Task difficulty explained 16% of the variance of inter-trial change in pupil diameter (BCPD) vs. only 7% of the variance of the intra-trial measure (CPD). Inter-trial change in pupil diameter appears to be more sensitive to task difficulty than intra-trial change in pupil diameter.

The better ability of the BCPD to distinguish task difficulty is likely due to its use of the mean of an entire (control) task as the baseline. Recall that the control task was performed before both easy and difficult tasks. This construction of the BCPD measure affords a more straightforward interpretation since no mental task was required from participants during the control task. Meanwhile, the use of a short, fixed time segment during the beginning of a trial as the baseline for the construction of the CPD, such as the use of a 2-second window as in this study, may lead to misleading statistical outcomes, e.g., all CPD outcomes were < 0. Such outcomes may then be mistakenly interpreted as an increase in pupil diameter constriction over the entire duration of the task. Instead, the pupil constricts during the 3 minutes of the task relative to the the first 2 seconds of the task. Constriction is slower during the more difficult task, but the interpretation of the CPD metric is still somewhat difficult due to the shortness of the time window used as baseline.

During the first 2 seconds of each trial, pupil diameter increased. Either the task itself caused a delayed constriction relative to the first 2 seconds, or the first 2 seconds may have induced arousal related to task novelty. Simon [59] noted that sudden intense stimuli can produce large effects on the autonomic nervous system, e.g., arousal. More recent neuropsychological studies show evidence of enhanced activation of frontal brain regions, e.g., the anterior cingulate cortex [60, 61] in reaction to novel stimuli. We thus caution against using intra-trial baseline differencing and advocate inter-trial pupillary measures, although we reiterate that off-axis pupil distortion should be taken into account.

Interestingly, none of the present analyses of microsaccadic or pupillary responses to task difficulty revealed any significant effect of working memory capacity (WMC). Past work has reported significant relationships between working memory capacity and performance of various complex cognitive tasks e.g., reading comprehension, and reasoning [49, 50]. Such findings suggest that participants with high WMC should experience lower cognitive load in difficult tasks than individuals with low working memory capacity. Indeed, our results support this type of relation between WMC and response accuracy of mental arithmetic tasks. Participants with high working memory capacity appear to have sufficient resources to meet cognitive demands of more difficult tasks, which is consistent with Sweller’s [1] original production-system model of cognitive load. Difficult task demands may have exceeded the resources of low working memory individuals causing a decrease in task accuracy.

Despite the moderating role of working memory capacity on the relation between cognitive load and pupillary responses [48], the relation between microsaccade activity and working memory capacity is scarcely found in the literature. Moreover, Kang & Woodman [62] concluded that microsaccadic and saccadic gaze shifts do not provide a sensitive measure of memory storage. In line with the literature, we demonstrated that neither of the main effects of WMC nor of interaction were significant in the analysis of covariance of microsaccadic or pupillary measures. We may conclude that the lack of effects of WMC potentially bolsters the remaining analyses suggesting that microsaccade magnitude and change in pupil diameter are sensitive to task difficulty independent of working memory capacity.

The direct comparison of microsaccadic and pupillary task-evoked measures revealed their ability to discriminate both difficult and easy mental tasks from the control task. The results of multinomial logistic regression analysis showed that both inter-trial change of pupil diameter and microsaccade magnitude were able to differentiate between tasks at a statistically significant level.

To summarize, our study corroborated earlier work showing that microsaccade magnitude may serve as a reliable and sensitive discriminant of task difficulty, *vis-à-vis* cognitive load. We also corroborate earlier classic work regarding pupil diameter, showing in particular that inter-trial change in pupil diameter (BCPD) may also serve as a comparable indicator.

## 6 Limitations

We caution that although the BCPD measure appeared to be reliable and sensitive, it suffers from a serious limitation in that it requires the eye to be held still and on-axis with respect to the eye tracking camera. This defeats the purpose of using an eye tracker in the first place. Allowing the eye to move off-axis will lead to potentially incorrect manifestation of pupil diameter due to its appearance as an ellipse. As far as we know, allowing the eyes to move freely should have no effect on microsaccadic magnitude, making it potentially more useful and robust in terms of ecological validity. Unlike pupil diameter, microsaccade magnitude should also be free from the influence of ambient light. We thus advocate microsaccade magnitude as the potentially more reliable, non-invasive, measure of cognitive load, also possibly capable of real-time implementation. Such real-time time applications could, for example, be used as a means of reducing interruption costs if notifications can be delivered at moments of lower mental workload during interactive task execution, e.g., as shown by Bailey & Iqbal [63].

We advocate microsaccadic response with a modicum of caution, however, as its chief limitation is sampling rate, which necessarily needs to be high (≥300 Hz) in order to be able to capture the shortest of microsaccades. We should be also aware that future experiments are needed to investigate the response of microsaccade magnitude to eye movements, light conditions, as well as sampling rates.

## 7 Conclusions

We briefly reviewed cognitive load and its connection to task-evoked eye movement measures: pupillary and microsaccadic responses. We summarized methods of estimating cognitive load as thought to be influenced by task difficulty: A) by obtaining the averaged difference in pupil diameter with respect to inter- or intra-trial (averaged) baseline, and B) by obtaining measures related to microsaccades, namely their mean magnitude and rate of occurrence. With respect to microsaccade magnitude, but not microsaccade rate, our findings corroborate those of Siegenthaler et al. [19] and extend their work by directly comparing microsaccade metrics with pupillometric measures. We have discussed the limitations of pupillometric measures and advocated measurement of microsaccadic activity as a more viable alternative for estimation of task difficulty *vis-à-vis* cognitive load. Being able to distinguish a user’s level of cognitive load, especially in real-time, has significant implications for design and/or evaluation of interactive systems. To the best of our knowledge our study is the first to directly compare reliability and sensitivity of task-evoked pupillary and microsaccadic measures of cognitive load.

## Supporting information

S1 File This dataset contains the main characteristics of microsaccades for microsaccade main sequence analysis. The data were used to prepare the Fig 5.
(CSV)

S2 File This dataset contains results of NASA-TLX and digit SPAN tests along with the trial-wise averaged main microsaccadic and pupil dilation measures. The dataset was used to prepare the Figs 4, 6 and 7.
(CSV)

S3 File This dataset is for reliability analysis of the NASA task load index (NASA-TLX). The dataset was used for reliability analyses of NASA-TLX scale within the present study.
(CSV)

S4 File The document contains supporting information on all datasets which were used in the analyses of results presented in the article “Eye tracking cognitive load using pupil diameter and microsaccades with fixed gaze”. The document is structured file by file with description of each dataset and each variable they contain with their value labels (if applicable).
(PDF)
