# Supplementary material for: Eye tracking cognitive load using pupil diameter and microsaccades with fixed gaze
Source: PLoS One. 2018 Sep 14;13(9):e0203629. doi: 10.1371/journal.pone.0203629 (PMC6138399; doi:10.1371/journal.pone.0203629)
Supplement: S4 File — The document is structured file by file with description of each dataset and each variable they contain with their value labels (if applicable). (PDF) [file pone.0203629.s004.pdf]

Datasets Supporting Information
for the Manuscript [PONE-D-18-12860] "Eye Tracking Cognitive Load Using Pupil Diameter and Microsaccades with Fixed Gaze"

Krzysztof Krejtz, Andrew T. Duchowski, Anna Niedzielska, Cezary Biele, and Izabela Krejtz

August 16, 2018

The document contains the supportive information on all datasets which were used in the analyses of results presented in the article "Eye Tracking Cognitive Load Using Pupil Diameter and Microsaccades with Fixed Gaze". The document is structured file by file with description of each dataset and each variable they contain with their values labels (if applicable).

1 Data Dictionary for Dataset "msac.csv"

Description: A microsaccades-wise dataset containing main characteristics of microsaccades for microsaccade main sequence analysis. The dataset contains the following variables.

subj A unique subject ID

block A unique block of trials ID factor. Values from 0 to 5 indicating 6 subsequent blocks of trials in the experimental procedure.

ttype A trial type identifier factor with the following categories' values labels

- **DIFF** A Difficult task: count backwards by 17
- **EASY** An Easy task: count forward by 2
- **CONTROL** A control task

mag A microsaccade amplitude

amp A microsaccade peak velocity

dur A microsaccade duration

2 Data Dictionary for Dataset "data_stats.csv"

Description: A trial-wise dataset of NASA-TLX, results of Digit SPAN test along with the averaged on trials main microsaccadic and pupil dilation measures. The dataset contains the following variables.

subj A unique subject ID

block A unique block of trials ID factor. Values from 0 to 5 indicating 6 subsequent blocks of trials in the experimental procedure

ttype A trial type identifier factor with the following categories' values labels

mag Microsaccade amplitude averaged by trial

amp Microsaccade peak velocity averaged by trial

msrt Microsaccade rate averaged by trial

pcpd Inter-Trial Percentage Pupil Diameter Change averaged by trial

bpcpd Intra-Trial Percentage Pupil Diameter Change averaged by trial

corr Proportion of correct responses in the block of trials

tlx A NASA-TLX averaged score

WM_mean A mean of forward (fTE_ML) and backward (bTE_ML) DSPAN - indicator of working memory capacity

fTE_ML Two-error maximum length, the traditional measure of a participant's FORWARD digit span. It is the last DIGIT SPAN a participant gets correct before making two consecutive errors.

bTE_ML Two-error maximum length, the traditional measure of a participant's BACKWARDS digit span. It is the last DIGIT SPAN a participant gets correct before making two consecutive errors.

wm A factor of median-split of mean working memory with values "HIGH" (above median) and "LOW" (below median)

3 Data Dictionary for Dataset "psych_demo.csv"

Description: A dataset for reliability analysis of NASA Task Load Index (NASA-TLX) scale. The dataset contains the following variables.

subj A unique subject ID

block A unique block of trials ID factor. Values from 0 to 5 indicating 6 subsequent blocks of trials in the experimental procedure.

ttype A trial type identifier factor with the following categories' values labels

- **DIFF** A Difficult task: count backwards by 17
- **EASY** An Easy task: count forward by 2
- **CONTROL** A control task

tlx1 Answer to NASA-TLX item "How mentally demanding was the task?" from 1 (Very low) to 21 (Very high)

tlx2 Answer to NASA-TLX item "How physically demanding was the task?" from 1 (Very low) to 21 (Very high)

tlx3 Answer to NASA-TLX item "How hurried or rushed was the pace of the task?" from 1 (Very low) to 21 (Very high)

tlx4 Answer to NASA-TLX item "How successful were you in accomplishing what you were asked to do?" from 1 (Very low) to 21 (Very high)

tlx5 Answer to NASA-TLX item "How hard did you have to work to accomplish your level of performance?" from 1 (Very low) to 21 (Very high)
